# Hallmark features of conventional BCS superconductivity in 2H-TaS_2_

**DOI:** 10.1038/s41598-025-24342-8

**Published:** 2025-10-23

**Authors:** Frank Elson, Ola Kenji Forslund, Rasmus Palm, Ahmed Alshemi, Mahmoud Abdel-Hafiez, Gediminas Simutis, Toni Shiroka, Debarchan Das, Elizabeth Blackburn, Jonas Weissenrieder, Yasmine Sassa, Martin Månsson

**Affiliations:** 1 Department of Applied Physics, KTH Royal Institute of Technology, 10691 Stockholm, Sweden; 2 Physik-Institut, Universität Zürich, Winterthurerstrasse 190, 8057 Zurich, Switzerland; 3 Department of Physics and Astronomy, Uppsala University, Box 516, 75120 Uppsala, Sweden; 4 Institute of Chemistry, University of Tartu, Ravila 14a, 50411 Tartu, Estonia; 5 Division of Synchrotron Radiation Research, Lund University, 22100 Lund, Sweden; 6 Department of Applied Physics and Astronomy, University of Sharjah, P.O. Box 27272, Sharjah, United Arab Emirates; 7 PSI Center for Neutron and Muon Sciences CNM, PSI, 5232, Villigen, Switzerland; 8 Department of Physics, Chalmers University of Technology, 41296 Göteborg, Sweden; 9 Laboratorium für Festkörperphysik, ETH Zürich, 8093 Zurich, Switzerland

**Keywords:** Van der Waals, Superconductivity, Muon spin rotation *μ*^+^SR, Materials science, Physics

## Abstract

Layered transition metal dichalcogenides (TMDs) are model systems to investigate the interplay between superconductivity and the charge density wave (CDW) order. Here, we use muon spin rotation and relaxation (*μ*^+^SR) to probe the superconducting ground state of polycrystalline 2H-TaS_2_, which hosts a CDW transition at 76 K and superconductivity below 1 K. The *μ*^+^SR measurements, conducted down to 0.27 K, are consistent with a nodeless, BCS-like single-gap *s*-wave state. Fits to the temperature dependence of the depolarization rate and Knight shift measurements support spin-singlet pairing. Crucially, no evidence of time-reversal symmetry breaking (TRSB) is observed, distinguishing 2H-TaS_2_ from polymorphs like 4Hb-TaS_2_, where TRSB and unconventional superconductivity have been reported. These findings establish 2H-TaS_2_ as a canonical BCS superconductor and provide a reference point for understanding the diverse electronic ground states that emerge in structurally distinct TMD polymorphs.

## Introduction

In recent years, layered van der Waals (vdW) materials have emerged as ideal platforms to explore novel quantum phenomena, including unconventional superconductivity, magnetism, topological states, and competing charge density wave (CDW) orders^1,2^. Among these materials, transition metal dichalcogenides (TMDs) of the form MX_2_ (M = transition metal, X = chalcogen) stand out due to their rich interplay between superconductivity and CDW phases, often manifesting in phase diagrams which depend critically on external tuning parameters such as pressure, doping, and dimensionality^3–5^. This interplay is exemplified in systems like 1T-TiSe_2_, NbSe_2_, and NbTe_2_, where superconductivity often emerges upon suppression of CDW order, suggesting competition or coexistence of these phenomena^3–10^.

The compound 2H-TaS_2_ is one example of this family, characterized by a CDW transition at approximately $T_{\text {CDW}}= 76$ K and superconductivity below $T_{\text {C}}= 1$ K^3–5,11^. Earlier works have indicated potential anisotropy in its superconducting gap structure and hinted at unconventional pairing mechanisms^11^, raising the fundamental question of whether the observed superconductivity conforms strictly to conventional BCS theory^12^ or if deviations indicating unconventional behavior exist^4,5^. A particularly illustrative contrast can be drawn with the 1T polymorph of TaS_2_, which undergoes a cascade of CDW transitions culminating in the formation of a commensurate $\sqrt{13} \times \sqrt{13}$ superstructure at low temperatures^13^. This structural modulation opens a Mott gap driven by strong electronic correlations, rendering the system insulating despite its metallic origin^14^. This behavior strongly differs from the metallic and superconducting ground state observed in the 2H phase. Furthermore, 4Hb-TaS_2_, a structural hybrid composed of alternating 1T- and 1H-like layers, has recently been shown to exhibit time-reversal symmetry breaking (TRSB) in its superconducting state—an observation suggestive of unconventional, possibly chiral, pairing mechanisms^15,16^. While the mechanisms underlying the Mott insulating state in 1T-TaS_2_ and the unconventional superconductivity in 4Hb-TaS_2_ are becoming increasingly well understood, the microscopic nature of superconductivity in 2H-TaS_2_ remains unclear. Reports across TaS_2_ polymorphs and thicknesses reveal a diverse superconducting landscape. In monolayer 1H-TaS_2_, low-*T* STM/STS indicates a nodal superconducting state, with disorder driving it toward a conventional gapped *s*-wave state^17^. By contrast, bulk 2H-TaS_2_ STM/STS shows a gapped local densities of states (LDOS) and chiral charge order, with a distribution of gap values in the superconducting state^18^. Reducing thickness in 2H-TaS_2_ enhances $T_c$ relative to bulk^19^, and in engineered/re-stacked TaS_2_ nanosheets the in-plane upper critical field can exceed the Pauli limit^20^. Under hydrostatic pressure, $T_c$ is strongly enhanced as the CDW weakens^3,21^. Taken together, these results motivate a bulk, volume-sensitive probe such as muon spin rotation/relaxation (*μ*^+^SR) to test for nodeless vs. nodal behavior and to assess spin-singlet pairing in the 2H polytype.

In this study, we use *μ*^+^SR to investigate the superconducting state of polycrystalline 2H-TaS_2_ down to temperatures as low as *T* = 0.27 K, enabling direct observation of its superconducting gap symmetry and pairing state. Our measurements reveal clear evidence for a conventional *s*-wave gap symmetry, well described by standard BCS theory. This finding is supported by temperature-dependent Knight shift measurements indicating spin-singlet pairing. Crucially, we also confirm the absence of TRSB, clearly differentiating the superconducting properties of 2H-TaS_2_ from more complex polymorphs like 4Hb-TaS_2_^15,16^. Establishing that 2H-TaS_2_ is a conventional BCS superconductor is of foundational importance and holds implications for engineering layered materials in quantum technologies. It defines a baseline case within the TMD family, serving as a reference point for interpreting more exotic behaviors in related compounds. By clarifying the nature of superconductivity in 2H-TaS_2_, our findings contribute essential insight toward disentangling the intertwined roles of CDW order, electronic correlations, and multiband effects in layered quantum materials.

## Results

To investigate the superconducting properties of polycrystalline 2H-TaS_2_, we performed *μ*^+^SR measurements in both zero-field (ZF) and transverse-field (TF) configurations. These measurements provide microscopic insight into the superconducting gap symmetry, the internal magnetic field distribution, and the nature of the vortex state^22,23^. Below, we present a detailed analysis of the time spectra, depolarization rates, and extracted superconducting parameters.

### Zero-field and transverse-field *μ*^+^SR measurements

**Fig. 1:**
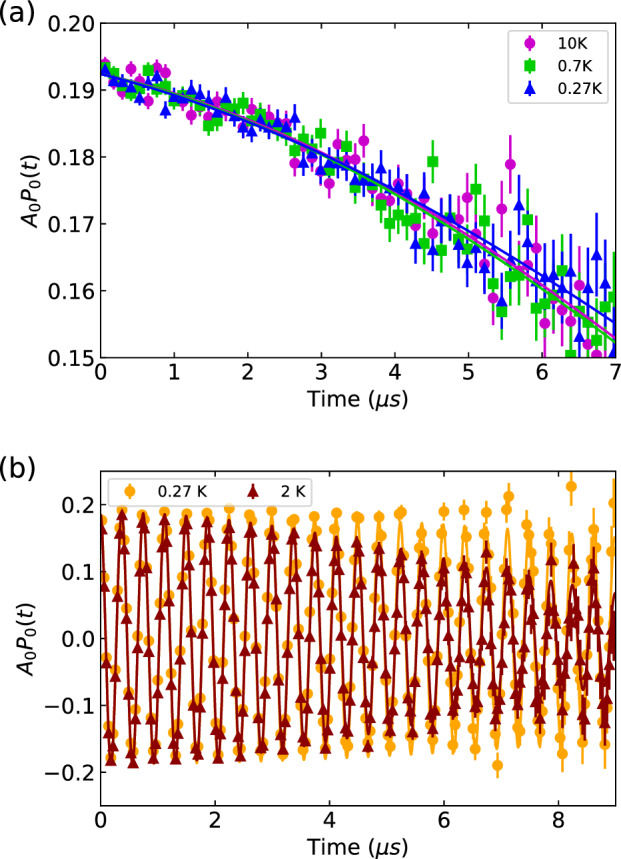
Muon spin rotation and relaxation (*μ*^+^SR) measurements on 2H-TaS_2_. (**a**) Zero-field (ZF) muon spin relaxation spectra at temperatures of 10 K (pink), 0.7 K (green), and 0.27 K (blue), demonstrating the absence of spontaneous magnetic fields. Solid lines represent fits using the Gaussian Kubo–Toyabe relaxation function. (**b**) Transverse-field (TF) muon polarization functions measured above (2 K, orange) and below (0.27 K, red) the superconducting transition temperature, under a transverse applied magnetic field of 200 G. Enhanced damping at 0.27 K indicates the formation of a superconducting vortex lattice.

Figure 1a shows the muon time spectra taken in ZF at T = 10 K, 0.7 K, and 0.27 K. The data are fitted using:

1 $$\begin{aligned} A_0P(t) & = A_s e^{-\lambda t} \left( \frac{1}{3} + \frac{2}{3}\left[ 1-\left( \sigma t\right) ^2 e^{-\frac{1}{2} (\sigma t)^2}\right] \right) \\ & = A_s e^{-\lambda t} \text {GKT}(t) \end{aligned}$$

where $A_s$ is the sample asymmetry, $\sigma$ is the depolarisation of the muon, and GKT(*t*) is the static Gaussian Kubo-Toyabe function. The GKT(*t*) function models the depolarization of muon spins in a system with randomly oriented, static local magnetic fields–typically arising from nuclear dipole moments—and is commonly used in ZF *μ*^+^SR to identify the presence or absence of spontaneous internal magnetic fields. The ZF muon time spectra show no discernible difference across temperatures and no coherent oscillations below $T_c$, indicating the absence of magnetic ordering.

Figure 1b displays the transverse-field (TF) 2SR time spectra measured under an applied magnetic field of $\mu _0$H = 200 G at two temperatures: 0.27 K and 2 K, corresponding to below and above the superconducting transition temperature respectively. The data is fitted using a Gaussian relaxing cosine function:

2 $$\begin{aligned} A_0P(t) = A_s e^{-\frac{1}{2} (\sigma t)^2} \cos \left( 2\pi \nu t + \frac{\pi \phi }{180} \right) \end{aligned}$$

where $A_s$ is the sample asymmetry, $\sigma$ is the muon depolarization rate, $\nu$ the frequency of oscillation, and $\phi$ a phase offset. At T = 2 K, oscillations remain largely undamped due to a relatively uniform magnetic field distribution in the sample, while a significant damping is observed at 0.27 K, indicating a broader internal magnetic field distribution, consistent with the formation of the vortex lattice below $T_c$.

### Determination of the upper critical field $H_{c2}$

**Fig. 2:**
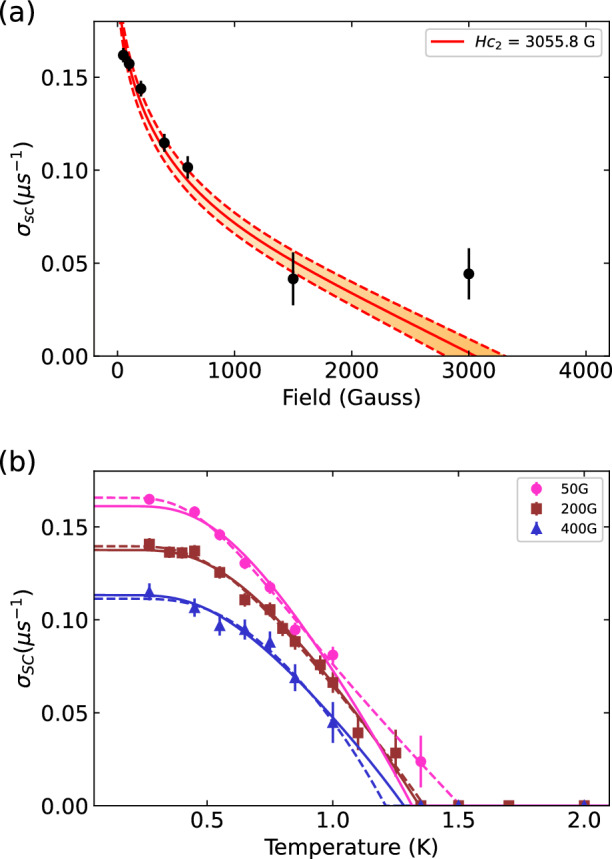
Field and temperature dependence of superconducting depolarization rate in 2H-TaS_2_. (**a**) Field dependence of the superconducting muon spin depolarization rate ($\sigma _{\textrm{SC}}$) measured at $T = 0.27$ K. The solid line represents a fit using Brandt’s formula, yielding an upper critical field ($H_{c2}$) of $3056 \pm 505$ G. The final data point at 3000 G was excluded due to saturation effects in the measured depolarization rates, indicating an experimental limitation rather than an intrinsic property. Shaded areas and dashed lines indicate the fitting uncertainty. (**b**) Temperature dependence of $\sigma _{\textrm{SC}}$ measured at applied fields of 50 G, 200 G, and 400 G. Dashed lines represent fits using a single-gap *s*-wave superconducting model, and the solid lines show fits according to the BCS model with fixed gap $\Delta (0)=1.764\,k_\textrm{B}T_\textrm{c}$. Values less than the nuclear contribution are set to zero.

Next, to quantify the upper critical field $H_{c2}$, we measured the muon depolarization rate $\sigma _T$ as a function of applied magnetic field *H*. Above $T_c$, the depolarization rate $\sigma _T$ reflects the field-independent contribution from the temperature-independent nuclear dipolar contribution ($\sigma _n$) mainly from Ta nuclei with $I^{181}{Ta} = 7/2$^24^. Below $T_c$, an additional depolarization component $\sigma _{sc}$ arises from the inhomogeneous field distribution of the superconducting vortex lattice. The total depolarization $\sigma _T$ is expressed as $\sigma _T^2 = \sigma _n^2 + \sigma {sc}^2$. By subtracting the nuclear contribution $\sigma _n$ (assumed to be constant in the restricted temperature range above $T_{\text{C}}$) to the total depolarization $\sigma _T$, we isolate the superconducting contribution $\sigma _{sc}$, which is used to extract $H_{c2}$ by fitting to the Brandt’s model^25^, described as:

3 $$\begin{aligned} \begin{aligned} \sigma _{sc}&= 4.83 \times 10^4 \left( 1-\frac{H}{H_{c2}}\right) \times \left[ 1+1.21\left( 1-\sqrt{\frac{H}{H_{c2}}}\right) ^3\right] \lambda ^{-2} \end{aligned} \end{aligned}$$

where *H* is the applied field, $\lambda$ the London penetration depth, and $H_{c2}$ the fitted upper critical field. Figure 2a shows $\sigma _{sc}(H)$ at T = 0.27 K fitted to Brandt’s formula, yielding $H_{c2} = 3055.8 \pm 505.4$ G.

### Determination of the gap symmetry from temperature-dependent depolarization

We next analyze the temperature-dependent TF $\mu ^+$SR measurements performed at applied fields of $\mu _0$H = 50 G, 200 G, and 400 G (Fig. 2b). Below $T_c$, the superconducting depolarization rate $\sigma _{sc}$ increases with decreasing temperature and saturates around $T \approx 0.5$ K. This increase in $\sigma _{sc}$ reflects the gradual build up of superfluid density as the superconducting state develops. Since $\sigma _{sc}$ is directly proportional to the *q*-integrated superfluid density, its temperature dependence provides valuable information about the symmetry and structure of the superconducting energy gap. Thus, each data set was fitted to:

4 $$\begin{aligned} \sigma _{sc} &= 1 + \frac{1}{\pi } \int _{0}^{2\pi }\int _{\Delta _k}^{\infty } \frac{E}{\sqrt{E^2-{\Delta _k}^2}}\frac{\partial f}{\partial E} \,dE d\phi , \end{aligned}$$

where $f = \left[ 1 + \exp {E/k_B T}\right]$ is the Fermi function and $\Delta _k = \Delta (T)g(\phi )$ is the temperature and angular dependence of the superconducting gap. For an *s*-wave symmetry, there is no *q*-dependence on the gap, reducing down the expression to

5 $$\begin{aligned} \sigma _{sc} = 1 - \frac{1}{2k_B T } \int _{0}^{\infty } \cosh ^{-2}\left( \frac{\sqrt{\varepsilon ^2 + {\Delta (T)}^2}}{ 2k_B T} \right) \,d\varepsilon . \end{aligned}$$

with $\Delta (T)=\Delta (0)\tanh {\left[ 1.821\times 1.018(T_c / T -1)^{0.51}\right] }$ from the BCS theory^26^, and $\Delta (0)$ the size of the superconducting gap at zero temperature.

The dashed lines in Fig. 2b correspond to fits using a single-gap *s*-wave model, where both the gap magnitude $\Delta (0)$ and the critical temperature $T_c$ are treated as free parameters. In contrast, the solid lines represent fits based on the BCS prediction, where $\Delta (0)$ is fixed to the weak-coupling value $\Delta (0) = 1.764k_B T_c$. The results are summarized in Table 1. For $\mu _0$H = 200 G and 400 G, both approaches yield consistent results, supporting the applicability of BCS theory. While the fit at $\mu _0$H = 50 G shows a modest deviation, likely due to limited data points near $T_c$, the BCS model still provides a reasonable description of the data. Overall, the analysis strongly suggests that superconductivity in 2H-TaS$_2$ is well described by conventional BCS theory with a fully open *s*-wave gap. Albeit our powder-averaged $\mu ^+$SR data are well described by a single, nodeless $s$-wave gap, we cannot exclude weak anisotropy or closely spaced multi-gap scales that would yield a similar temperature dependence of $\sigma _{\textrm{sc}}(T)$ within our experimental uncertainty and base temperature (0.27 K $\approx 0.2T_c$). We therefore describe the superconducting gap as nodeless, BCS-like $s$-wave rather than strictly isotropic.

**Table 1:** Superconducting gap parameters and critical temperatures for 2H-TaS_2_.

H (G)	Δ(0) (meV)		T_*c*_ (K)	
	Fitted gap	BCS	Fitted gap	BCS
50	0.168 ± 0.004	0.187 ± 0.008	1.508 ± 0.019	1.315 ± 0.0589
200	0.178 ± 0.006	0.190 ± 0.002	1.394 ± 0.037	1.345 ± 0.016
400	0.194 ± 0.016	0.181 ± 0.004	1.210 ± 0.069	1.280 ± 0.031

Summary of the superconducting gap magnitudes $\Delta (0)$ and transition temperatures $T_c$ obtained from transverse-field *μ*^+^SR data at magnetic fields of 50 G, 200 G, and 400 G. The experimental data were analyzed using both a single-gap *s*-wave model (‘Fitted Gap’) and a conventional BCS model (‘BCS’) with the gap ratio fixed to $\Delta (0) = 1.764\,k_{\textrm{B}}T_c$.

### Knight shift analysis

Knight shift measurements further support this interpretation and reveal the superconducting pairing symmetry. The Knight shift reflects the local magnetic environment experienced by implanted muons and can be extracted from the temperature-dependent TF $\mu ^+$SR measurements. It is defined as the normalized shift in the internal magnetic field $H_s$ from the applied field $H_0$ as $K = \frac{H_{0} - H_{s}}{H_{s}}$.

In superconductors, the Knight shift provides a sensitive probe of the spin state of the Cooper pairs. For spin-triplet pairing ($S = 1$), the spins of the paired electrons remain aligned with the applied magnetic field, and the Knight shift therefore remains nearly constant across the superconducting transition. In contrast, for spin-singlet pairing ($S = 0$), the formation of pairs with zero net spin suppresses the spin susceptibility below $T_c$, leading to a measurable decrease in the Knight shift. The experimentally measured Knight shift comprises a temperature-dependent spin contribution, $K_s(T)$, and a temperature-independent orbital (or chemical) contribution, $K_{\textrm{orb}}$. Consequently, the total shift does not vanish as $T \rightarrow 0$ even for an ideal spin-singlet superconductor. The observed reduction of the total Knight shift below $T_c$ therefore reflects the expected suppression of $K_s(T)$, while the finite residual value at low temperature arises from $K_{\textrm{orb}}$, demagnetization effects, and field inhomogeneity in the vortex state.

**Fig. 3:**
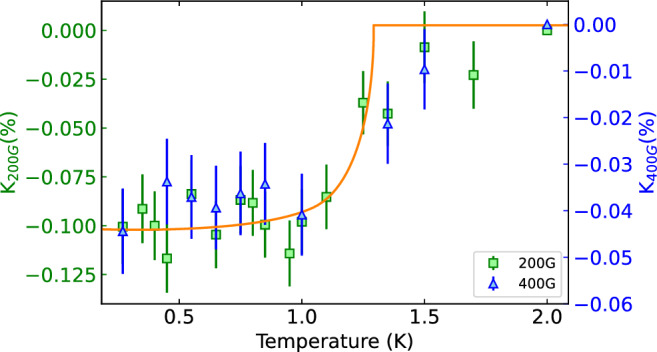
Temperature dependence of the Knight shift in 2H-TaS_2_. Knight shift measurements at applied fields of 200 G (green squares, left axis) and 400 G (blue triangles, right axis). A clear reduction in the Knight shift is observed below the superconducting transition temperature, consistent with spin-singlet pairing of Cooper pairs. The solid line is a guide to the eye illustrating the trend.

Figure 3 displays the Knight shift as a function of temperature at applied fields of $\mu _0$H = 200 G and 400 G. In both cases, a clear decrease is observed below the superconducting transition temperature, consistent with the formation of spin-singlet Cooper pairs (S = 0), as the lack of degeneracy in the Cooper pairs allows them to adapt to the magnetic field, leading to a decrease in field below T$_c$. This behavior further corroborates the conclusions drawn from the gap symmetry analysis and strongly supports a conventional BCS mechanism governing superconductivity in 2H-TaS_2_, as we see no signs of *d*-wave superconductivity in the temperature dependence of $\sigma _{sc}$.

### London penetration depth and Uemura analysis

**Fig. 4:**
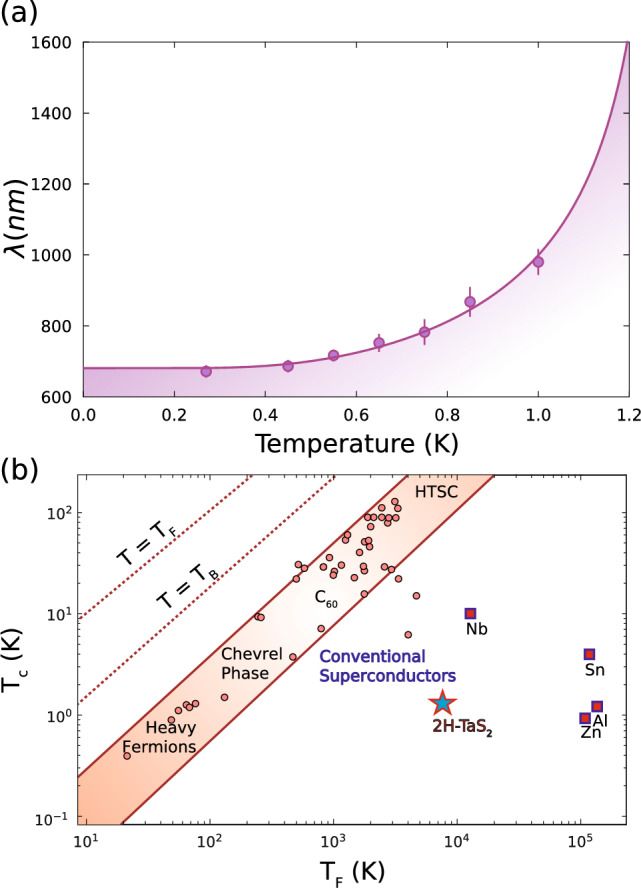
London penetration depth and Uemura plot analysis of 2H-TaS_2_. (**a**) Temperature dependence of the London penetration depth ($\lambda$), extracted from transverse-field muon spin rotation measurements. The solid line represents a fit based on the BCS superconducting model, illustrating conventional superconductivity. Shaded area indicates the uncertainty in the fit. (**b**) Uemura plot illustrating the superconducting transition temperature ($T_\textrm{c}$) versus the Fermi temperature ($T_\textrm{F}$) for various superconductors. The position of 2H-TaS_2_ (marked by a blue star) indicates its placement among conventional superconductors. Adapted from Ref.^27^.

To further characterize the superconducting state, we examine the London penetration depth, $\lambda$, which provides a measure of how deeply magnetic fields can penetrate into a superconductor. It is directly related to the superfluid density and thus serves as another sensitive probe of gap symmetry and pairing strength. In TF $\mu ^+$SR, the penetration depth can be inferred from the field dependence of the superconducting muon depolarization rate $\sigma _{sc}$. Because $\sigma _{sc}$ is affected by both the applied magnetic field and the vortex lattice structure, we extract the field-independent $\lambda$ by analyzing the temperature-dependent $\sigma _T$ values obtained at $\mu _0$H = 50 G, 200 G, and 400 G using Brandt’s model. The results are presented in Fig. 4a, yielding a zero-temperature penetration depth of $\lambda (0) = 678.3 \pm 4.8$ nm.

To contextualize, we extract the electronic parameters of 2H-TaS_2_ to find the Fermi temperature, $T_F$, and placing 2H-TaS_2_ onto the Uemura plot (Fig. 4b). The Sommerfeld coefficient, the electronic coefficient for scaling the temperature dependence of the heat capacity ($C = \gamma _n T$), is defined through the following equation;

6 $$\begin{aligned} \gamma _n = \left( \frac{\pi }{3}\right) ^{2/3} \frac{k_B^2 m^{*} n^{1/3}}{\hbar ^2} V_m \end{aligned}$$

where $m^*$ is the effective mass of the quasiparticles, *n* is the density of charge carriers per cubic meter, and $V_m$ is the molar volume of the material (molar mass/density)^28^. Rearranging this equation to find $m^*$, and using a Sommerfeld coefficient of $\gamma _n = 8.8$ mJ mol^−1^ K^−2^^3^, a carrier density of $2.39\times 10^{28}$ m^−3^ (found from the hall coefficient $R_H = 2.2 \times 10^{-4}$ cm^3^ C^−1^^29^, and using the relation $R_H = 1/ne$ where *e* is the electron charge), and a molar volume of $3.46 \times 10^{-5}$ m^3^ mol^−1^ (using a molar mass of $245.08$ g mol^−1^ and a density of 7090 kg m^−3^^30^), we find an effective mass of $m^* = 4.72 \times 10^{-30}\,{\text{kg}} = 5.18 m_e$ (where $m_e$ is the mass of the electron). We can then further use the relation

7 $$\begin{aligned} T_F = \frac{\hbar ^2 \left( 3\pi ^2 n\right) ^{2/3}}{2 m^* k_B} \end{aligned}$$

to find the Fermi temperature, $T_F$. Using the above values, we find a Fermi temperature of $T_F = 7599.44$ K. This is shown on the Uemura plot in Fig. 4b. As we can see, 2H-TaS_2_ sits towards the conventional superconductors side of the Uemuera plot.

## Discussion

Our $\mu ^+$SR measurements establish that the superconducting ground state of 2H-TaS_2_ exhibits all the hallmark features of a conventional BCS superconductor. Specifically, we identify a fully gapped *s*-wave order parameter, singlet pairing confirmed by suppression of the Knight shift, and an absence of TRSB. These findings align with previous reports indicating conventional superconductivity in undoped 2H-TaS_2_^3–5^, resolving historical ambiguities regarding unconventional pairing mechanisms previously suggested by macroscopic measurements^11^. Such discrepancies may originate from extrinsic sample quality variations or directional averaging effects inherent to different experimental methodologies.

**Fig. 5:**
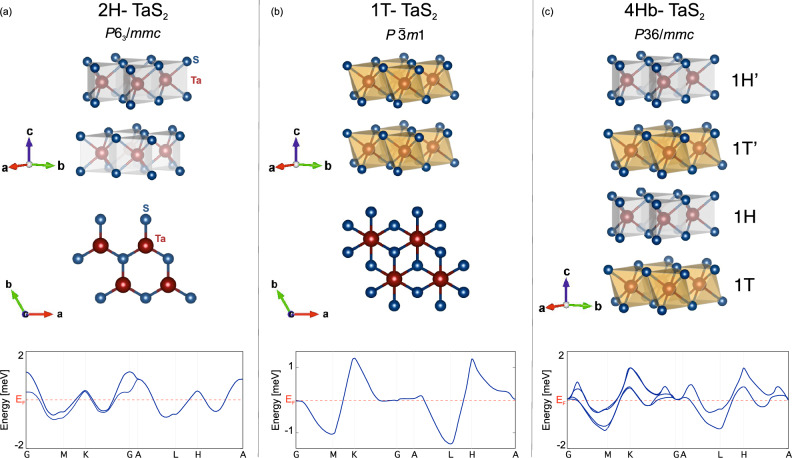
Crystal structures and schematic electronic band structures of TaS_2_ polymorphs. (**a**) Crystal structure (space group $P6_3/mmc$) and schematic band structure of the 2H-TaS_2_ polymorph. The trigonal prismatic coordination results in broad dispersive bands, isotropic electronic properties, and weak electronic correlations, facilitating conventional superconductivity. (**b**) Crystal structure (space group $P\bar{3}m1$) and schematic electronic band structure of the 1T-TaS_2_ polymorph. The octahedral coordination environment around Ta atoms leads to strong electronic correlations, narrow bands, and a prominent CDW state, driving the system toward a Mott insulating ground state. (**c**) Crystal structure (space group $P\bar{3}6/mmc$) and calculated band structure of the 4Hb-TaS_2_ polymorph, consisting of alternating layers (1T, 1T′, 1H, 1H′) creating structural asymmetry. The resulting complex structure supports multiband superconductivity, local symmetry breaking, and unconventional pairing mechanisms, including chiral superconductivity with time-reversal symmetry breaking (TRSB). In the structural models, blue spheres represent S atoms, and red spheres represent Ta atoms. The dashed red lines in the band structures indicate the Fermi level $E_\textrm{F}$.

A comparison with chemically doped variants, such as Cu-intercalated TaS_2_^5^, highlights the sensitivity of superconductivity to chemical doping, suggesting possible transitions from conventional to unconventional superconductivity. Structural and electronic considerations illustrated in Fig. 5 clearly depict why the physics of TaS_2_ polymorphs differ significantly. The 1T polymorph (Fig. 5b) undergoes sequential CDW transitions leading to a low-temperature commensurate CDW state with a $\sqrt{13}\times \sqrt{13}$ reconstruction of the Ta lattice^13^. This structural modulation significantly narrows the electronic bandwidth, producing flat electronic bands (Fig. 5b, bottom) and strong nesting vectors that localize electrons and drive the material into a Mott insulating state characterized by strong electronic correlations^14^, thus suppressing superconductivity.

Conversely, the hybrid 4Hb-TaS_2_ polymorph (Fig. 5c) exhibits unconventional superconductivity characterized by TRSB^15,16^. Structurally, 4Hb-TaS_2_ consists of alternating layers with 1T and 1H motifs, forming a natural heterostructure^31^. This stacking pattern creates an asymmetric interlayer environment that facilitates interband hybridization, charge transfer, and local symmetry breaking—conditions to stabilize multiband, chiral superconducting states. The complex band structure at the Fermi level of 4Hb-TaS_2_ (Fig. 5c, bottom) exhibits both dispersive and localized states, essential for unconventional superconductivity. Theoretical models suggest chiral $d+id$ or $p+ip$ pairing states emerge from sufficient interband coupling and Fermi surface frustration^32,33^. Moreover, local inversion symmetry breaking within the unit cell can lift degeneracies, promoting TRSB.

In 2H-TaS_2_ (Fig. 5a), uniform trigonal prismatic stacking maintains both global and local inversion symmetry, yielding broad dispersive Ta *d*-bands (Fig. 5a, bottom). This structural and electronic regularity corresponds well with the conventional superconductivity and absence of TRSB observed in our study. Therefore, our findings reinforce the notion that TRSB is not an intrinsic characteristic of TaS_2_ superconductors but rather emerges under specific structural and electronic conditions.

The distinct superconducting behaviors among TaS_2_ polymorphs exemplify a nuanced interplay between superconductivity and CDW phenomena, governed by structural motifs, electronic band structures, and correlation effects. In 2H-TaS_2_, a moderate CDW transition coexists harmoniously with conventional isotropic *s*-wave BCS superconductivity. Conversely, in 1T-TaS_2_, intense CDW-driven lattice distortions and strong electron-electron correlations suppress superconductivity, stabilizing instead a Mott insulating state. The intermediate scenario in 4Hb-TaS_2_–characterized by structural asymmetry, multiband interactions, and pronounced CDW order–fosters complex electronic interactions conducive to unconventional superconductivity with chiral pairing and TRSB. These observations highlight the critical role of structural and electronic correlations in determining the nature of superconductivity in layered quantum materials.

Collectively, the TaS_2_ family illustrates how subtle structural and electronic perturbations in layered quantum materials yield fundamentally different electronic ground states, ranging from Mott insulating (1T) to conventional superconducting (2H) and chiral superconducting (4Hb) phases. This polymorphic diversity provides an ideal platform to study exotic pairing mechanisms and correlated phenomena. Our results support the view that bulk 2H–TaS_2_ exhibits conventional, nodeless BCS-like superconductivity. Given the powder-averaged nature of $\mu ^+$SR and our base temperature, our measurements are insensitive to gap anisotropy or closely spaced multi-gap scales. Nevertheless, these insights hold broader implications for other layered materials, such as kagome metals^34,35^, twisted bilayer graphene^2^, and iron-based superconductors^36^, where minor structural modifications can induce transitions between conventional and topological phases.

Future theoretical investigations into interlayer hybridization and symmetry analyses will further illuminate the essential conditions required for TRSB and guide the rational design of novel artificial heterostructures aimed at achieving targeted superconducting properties.

## Methods

### Sample preparation

Polycrystalline samples of 2H-TaS_2_ were synthesized via chemical vapor transport (CVT) using elemental tantalum and sulfur, with sulfur vapor acting as the transport medium. Details of the synthesis protocol are available in Ref.^37^. The electrical and magnetic properties of the resulting 2H-TaS_2_ samples have been previously characterized in Refs.^3,4^. Resistivity and magnetic susceptibility measurements confirm a superconducting transition temperature of $T_c = 1$ K.

### The $\mu ^+SR$ experiments

Muon spin rotation/relaxation ($\mu ^+$SR) experiments were carried out at the Dolly instrument—part of the $\pi$E1 beamline—at the Paul Scherrer Institute (PSI) in Villigen, Switzerland. All $\mu ^+$SR data were collected in a field-cooled (FC) protocol to ensure homogeneous flux penetration during the superconducting state measurements. The powder sample was pressed into a dense pellet and mounted on a high-purity copper foil using Apiezon N grease to ensure thermal and mechanical stability. The sample was further secured with Kapton tape to minimize motion during cool down and measurement. The thin copper foil has a low muon stopping fraction and hence its contribution to asymmetry can be neglected (the muons missing the sample do not stop in the thin copper foil). Additional background suppression was achieved through the use of a veto detector, which filters out muons that miss the sample and reduces spurious signal contributions from surrounding components. All measurements were performed under high-vacuum, cryogenic conditions, with the temperature varied between T = 10 K and 0.27 K to probe the normal and superconducting states of the sample.

### Crystal structures and band structure calculations

Crystal structure visualizations were created using VESTA software^38^, and the electronic band structures were adapted from the Materials Project database^39^. Details about the band structure calculations are found in refs.^40,41^. Spin–orbit coupling (SOC) originating from the Ta 5*d* orbitals is substantial in magnitude but, in the centrosymmetric 2H-polytype, its symmetry-allowed effects are moderate: SOC preserves Kramers degeneracy and does not lift spin degeneracy at the Fermi surface. Nevertheless, it can mix orbital and spin characters of the electronic states, giving rise to a temperature-independent orbital contribution to the Knight shift. The observed reduction in the total Knight shift below $T_c$ thus reflects suppression of the spin susceptibility (consistent with spin-singlet pairing), while the residual value at low temperature is attributed to $K_{\textrm{orb}}$ and demagnetization effects.

## Data Availability

The data supporting the findings of this study are available through https://musruser.psi.ch/. Analysis of the data is available from the corresponding authors on reasonable request.

## References

[1] Burch, K. S., Mandrus, D. & Park, J.-G. Magnetism in two-dimensional van der Waals materials. *Nature* **563**, 47–52. 10.1038/s41586-018-0631-z (2018). 30382199 10.1038/s41586-018-0631-z

[2] Cao, Y. et al. Correlated insulator behaviour at half-filling in magic-angle graphene superlattices. *Nature* **556**, 80. 10.1038/nature26154 (2018). 29512654 10.1038/nature26154

[3] Abdel-Hafiez, M. et al. Enhancement of superconductivity under pressure and the magnetic phase diagram of tantalum disulfide single crystals. *Sci. Rep.* **6**, 31824. 10.1038/srep31824 (2016). 27534898 10.1038/srep31824 PMC4989151

[4] Kvashnin, Y. et al. Coexistence of superconductivity and charge density waves in tantalum disulfide: Experiment and theory. *Phys. Rev. Lett.* **125**, 186401. 10.1103/PhysRevLett.125.186401 (2020). 33196259 10.1103/PhysRevLett.125.186401

[5] Wagner, K. E. et al. Tuning superconductivity in Cu_*x*_TaS_2_. *Phys. Rev. B* **78**, 104520. 10.1103/PhysRevB.78.104520 (2008).

[6] Joe, Y. I. et al. Emergence of charge density wave domain walls above the superconducting dome in 1T-TiSe_2_. *Nat. Phys.* **10**, 421–425. 10.1038/nphys2935 (2014).

[7] Cho, K. et al. Using controlled disorder to probe the interplay between charge order and superconductivity in NbSe_2_. *Nat. Commun.* **9**, 2796. 10.1038/s41467-018-05153-0 (2018). 30022110 10.1038/s41467-018-05153-0 PMC6052160

[8] Majumdar, A. et al. Interplay of charge density wave and multiband superconductivity in layered quasi-two-dimensional materials: The case of 2H-NbS_2_ and 2H-NbSe_2_. *Phys. Rev. Mater.* **4**, 084005. 10.1103/PhysRevMaterials.4.084005 (2020).

[9] Leroux, M. et al. Strong anharmonicity induces quantum melting of charge density wave in 2H-NbSe_2_ under pressure. *Phys. Rev. B* **92**, 140303. 10.1103/PhysRevB.92.140303 (2015).

[10] Jang, W.-J. et al. Direct observation of multiband charge density waves in NbTe_2_. *Phys. Rev. B* **106**, 125110. 10.1103/PhysRevB.106.125110 (2022).

[11] Nagata, S. et al. Superconductivity and CDW behavior in TaS_2_ compounds. *J. Phys. Chem. Solids* **53**, 1259–1263. 10.1016/0022-3697(92)90242-6 (1992).

[12] Bardeen, J., Cooper, L. N. & Schrieffer, J. R. Theory of superconductivity. *Phys. Rev.* **108**, 1175–1204. 10.1103/PhysRev.108.1175 (1957).

[13] Hovden, R. et al. Atomic lattice disorder in charge-density-wave phases of exfoliated dichalcogenides (1T-TaS_2_). *Proc. Natl. Acad. Sci.* **113**, 11420–11424. 10.1073/pnas.1606044113 (2016). 27681627 10.1073/pnas.1606044113 PMC5068312

[14] Wang, Y. D. et al. Band insulator to Mott insulator transition in 1T-TaS_2_. *Nat. Commun.*10.1038/s41467-020-18040-4 (2020). 32839433 10.1038/s41467-020-18040-4 PMC7445232

[15] Ribak, A. et al. Chiral superconductivity in the alternate stacking compound 4Hb-TaS_2_. *Sci. Adv.* **6**, eaax9480. 10.1126/sciadv.aax9480 (2020). 32258393 10.1126/sciadv.aax9480 PMC7101217

[16] Persky, E. et al. Magnetic memory and spontaneous vortices in a van der Waals superconductor. *Nature* **607**, 692–696. 10.1038/s41586-022-04855-2 (2022). 35896649 10.1038/s41586-022-04855-2

[17] Vaňo, V. et al. Evidence of nodal superconductivity in monolayer 1H-TaS_2_ with hidden order fluctuations. *Adv. Mater.* **35**, 2305409. 10.1002/adma.202305409 (2023). 10.1002/adma.202305409 37592888

[18] Guillamón, I. et al. Chiral charge order in the superconductor 2H-TaS_2_. *New J. Phys.* **13**, 103020. 10.1088/1367-2630/13/10/103020 (2025).

[19] Navarro-Moratalla, E. et al. Enhanced superconductivity in atomically thin TaS_2_ via in situ electric field tuning. *Nano Lett.* **20**, 2718–2725. 10.1021/acs.nanolett.9b05220 (2020).

[20] Ma, Y. et al. Unusual evolution of Bc2 and Tc with inclined fields in restacked TaS_2_ nanosheets. *NPJ Quantum Mater.* **3**, 34. 10.1038/s41535-018-0107-2 (2018).

[21] Freitas, D. C. et al. Strong enhancement of superconductivity at high pressures within the charge-density-wave states of 2H-TaS_2_ and 2H-TaSe_2_. *Phys. Rev. B* **93**, 184512. 10.1103/PhysRevB.93.184512 (2016).

[22] Suter, A. & Wojek, B. M. Musrfit: A free platform-independent framework for *μ*SR data analysis. *Phys. Procedia* **30**, 69–73. 10.1016/j.phpro.2012.04.042 (2012).

[23] Ghosh, S. K. et al. Recent progress on superconductors with time-reversal symmetry breaking. *J. Phys. Condens. Matter* **33**, 033001. 10.1088/1361-648X/abaa06 (2020). 10.1088/1361-648X/abaa06 32721940

[24] Jaeck, A. Tantalum-181. howpublished: https://www.chemlin.org/isotope/tantalum-181.

[25] Brandt, E. H. Properties of the ideal Ginzburg-Landau vortex lattice. *Phys. Rev. B* **68**, 054506. 10.1103/PhysRevB.68.054506 (2003).

[26] Gupta, R. et al. Isotropic *s*-wave superconductivity in the noncentrosymmetric charge density wave superconductor SePt_2_As_2_. *Phys. Rev. B* **102**, 144515. 10.1103/PhysRevB.102.144515 (2020).

[27] Sajilesh, K. P. et al. Investigations of the superconducting ground state of Zr_3_Ir: Introducing a new noncentrosymmetric superconductor. *Phys. Rev. Mater.* **3**, 104802. 10.1103/PhysRevMaterials.3.104802 (2019).

[28] Motla, K. et al. Superconducting and normal-state properties of the high-entropy alloy Nb-Re-Hf-Zr-Ti investigated by muon spin relaxation and rotation. *Phys. Rev. B* **105**, 144501. 10.1103/PhysRevB.105.144501 (2022).

[29] Naito, M. & Tanaka, S. Electrical transport properties in 2H-NbS_2_, -NbSe_2_, -TaS_2_ and -TaSe_2_. *J. Phys. Soc. Jpn.* **51**, 219–227. 10.1143/JPSJ.51.219 (1982).

[30] Johnston, D. & Keelan, B. Superconductivity and magnetism of M_x_(H_2_O)_y_TaS_2_ layered cointercalation compounds. *Solid State Commun.* **52**, 631–634. 10.1016/0038-1098(84)90722-1 (1984).

[31] Gao, J. J. et al. Origin of the large magnetoresistance in the candidate chiral superconductor 4Hb-TaS_2_. *Phys. Rev. B* **102**, 075138. 10.1103/PhysRevB.102.075138 (2020).

[32] Ganesh, R., Baskaran, G., van den Brink, J. & Efremov, D. V. Theoretical prediction of a time-reversal broken chiral superconducting phase driven by electronic correlations in a single TiSe_2_ layer. *Phys. Rev. Lett.* **113**, 177001. 10.1103/PhysRevLett.113.177001 (2014). 25379930 10.1103/PhysRevLett.113.177001

[33] Lüscher, B. E. & Fischer, M. H. *Superconductivity in a Chern Band: Effect of Time-Reversal-Symmetry Breaking on Superconductivity* **2506**, 16508 (2025).

[34] Yu, S.-L. & Li, J.-X. Chiral superconducting phase and chiral spin-density-wave phase in a Hubbard model on the Kagome lattice. *Phys. Rev. B* **85**, 144402. 10.1103/PhysRevB.85.144402 (2012).

[35] Wang, N. N. et al. Competition between charge-density-wave and superconductivity in the Kagome metal RbV_3_Sb_5_. *Phys. Rev. Res.* **3**, 043018. 10.1103/PhysRevResearch.3.043018 (2021).

[36] Fernandes, R., Chubukov, A. & Schmalian, J. What drives nematic order in iron-based superconductors?. *Nat. Phys.* **10**, 97. 10.1038/nphys2877 (2014).

[37] Chareev, D. A. et al. Stable sulfuric vapor transport and liquid sulfur growth on transition metal dichalcogenides. *Cryst. Growth Des.* **23**, 2287–2294. 10.1021/acs.cgd.2c01318 (2023). 37038405 10.1021/acs.cgd.2c01318 PMC10080655

[38] Momma, K. & Izumi, F. VESTA 3 for three-dimensional visualization of crystal, volumetric and morphology data. *J. Appl. Crystallogr.* **44**, 1272–1276. 10.1107/S0021889811038970 (2011).

[39] Jain, A. et al. Commentary: The materials project: A materials genome approach to accelerating materials innovation. *APL Mater.* **1**, 011002. 10.1063/1.4812323 (2013).

[40] Anubhav, J., Shyue, P. O., Geoffroy, H., Charles, M. & Jason, M. *Electronic Structure*. howpublished: https://docs.materialsproject.org/methodology/materials-methodology/electronic-structure/.

[41] Munro, J. et al. An improved symmetry-based approach to reciprocal space path selection in band structure calculations. *NPJ Comput. Mater.* **6**, 112. 10.1038/s41524-020-00383-7 (2020).

